# Deep learning denoising and deep learning–augmented contrast enhancement improve ultra-high-resolution photon-counting-detector CCTA image quality

**DOI:** 10.1186/s41747-026-00809-9

**Published:** 2026-11-13

**Authors:** Sebastian Steinmetz, Anton Kilburg, Antoine P Sanner, Andrea Kronfeld, Anna W. Grebe, Michaela Hell, Akos Varga-Szemes, Tilman Emrich, Tobias Bäuerle, Marc A. Brockmann, Ahmed E. Othman, Moritz C. Halfmann

**Affiliations:** 1 Department of Neuroradiology, University Medical Center Mainz, Johannes Gutenberg University, Mainz, Germany; 2 Department of Radiology, University Medical Center Mainz, Johannes Gutenberg University, Mainz, Germany; 3 Department of Cardiology, University Medical Center Mainz, Johannes Gutenberg-University, Mainz, Germany; 4 Division of Cardiovascular Imaging, Department of Radiology and Radiological Science, Medical University of South Carolina, Charleston, SC USA

**Keywords:** Cardiac imaging techniques, Coronary artery disease, Computed tomography angiography, Deep learning, Tomography (x-ray computed)

## Abstract

**Objective:**

Ultrahigh-resolution (UHR) coronary computed tomography angiography (CCTA) using a photon-counting detector (PCD) provides excellent spatial resolution but is limited by increased image noise. We evaluated whether deep learning (DL)-based denoising and contrast enhancement can further improve UHR-CCTA image quality.

**Materials and methods:**

UHR-CCTA scans from 25 patients with coronary artery disease were retrospectively analyzed. Images were reconstructed at 0.2-mm slice thickness without and with quantum iterative reconstruction (QIR, Q_off_–Q_4_), and subsequently processed using a DL tool for denoising and increasing iodine contrast enhancement. Subjective image quality (sharpness, noise, vessel wall delineation, and overall quality) was assessed by three radiologists using a 5-point Likert scale. Objective analysis included signal-to-noise ratio (SNR), contrast-to-noise ratio (CNR), and coronary wall sharpness. Differences between image sets were analyzed using Wilcoxon signed-rank tests.

**Results:**

DL-based CCTA demonstrated significantly superior subjective image quality across all parameters compared to standard reconstructions, with an overall median image quality ([interquartile interval, IQR]: 4 [3–5] *versus* 2 [2–3], *p* < 0.001). SNR and CNR were significantly higher for DL-based CCTA across all QIR levels (all *p* < 0.001). The highest values were observed for DL-based CCTAQ_4_ *versus* UHR-CCTAQ_4_ (SNR: 258.0 ± 147.5 *versus* 145.2 ± 50.1; CNR: 25.8 ± 17.1 *versus* 11.1 ± 4.5; both *p* < 0.001). Coronary wall sharpness was improved for DL-based CCTA for Q_1–4_ (all *p* < 0.027).

**Conclusion:**

DL-based denoising and iodine contrast enhancement effectively reduce noise and improve anatomic detail in UHR-CCTA, providing incremental value beyond vendor-specific reconstruction, particularly at high QIR strength levels.

**Key Points:**

- ***Question*** Deep learning reduces noise and enhances contrast in ultrahigh-resolution coronary CT angiography.
- ***Findings*** Image quality improves beyond standard iterative reconstruction methods, and vessel wall sharpness and diagnostic clarity significantly increase.
- ***Relevance statement*** This study aimed to determine at which levels of vendor-specific iterative reconstruction, DL-based denoising and contrast enhancement perform best in UHR CCTA, providing a foundation for future investigations on how these technical improvements may translate into diagnostic benefits.

**Graphical Abstract:**

**Figure:**
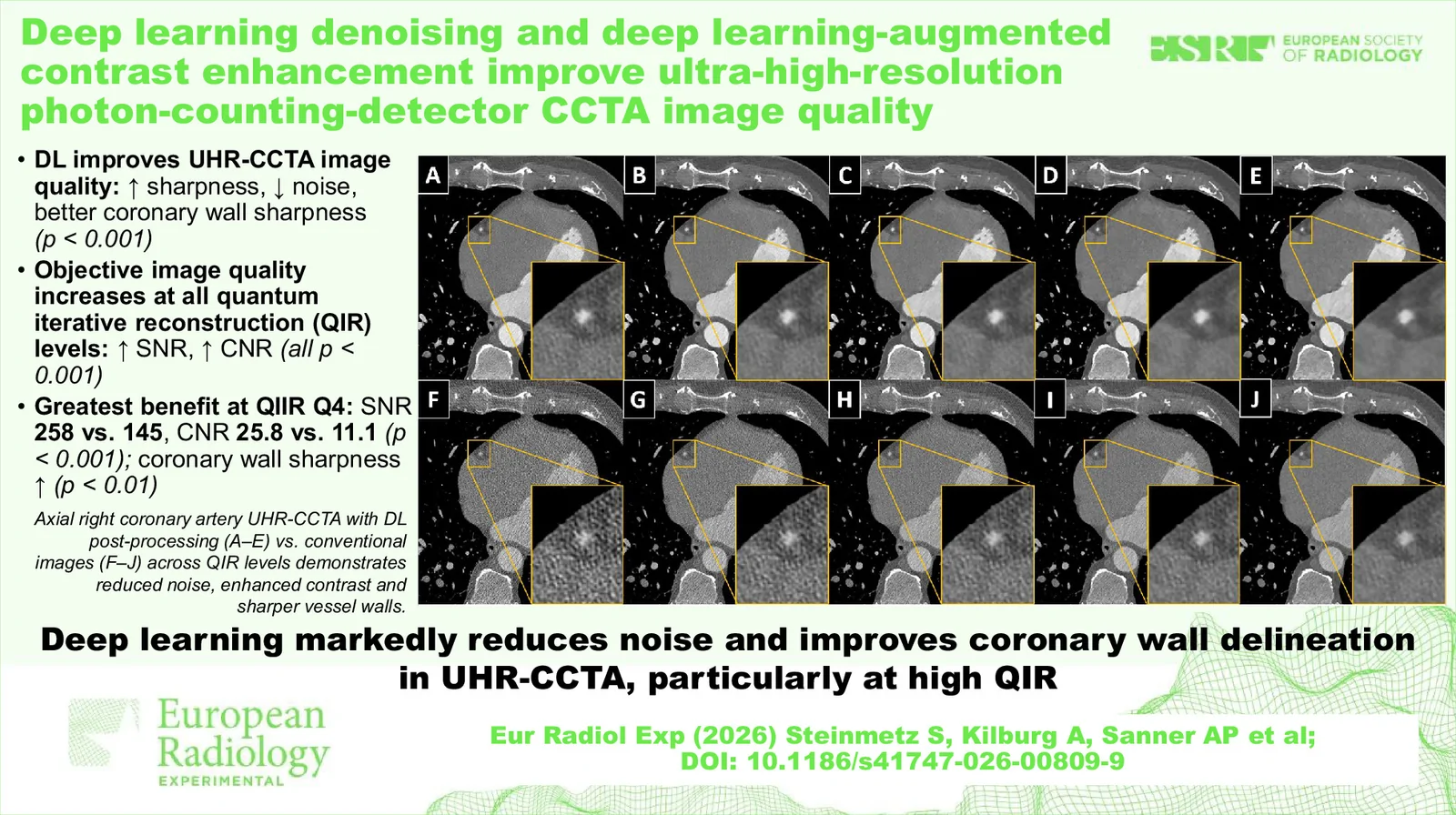

## Background

Artificial intelligence is increasingly transforming cardiovascular imaging. In the context of coronary artery disease (CAD), the leading cause of death worldwide, deep learning (DL)-based image post-processing may address the clinically challenging increase in image noise from recently emerged ultrahigh spatial resolution (UHR) coronary computed tomography angiography (CCTA) [1].

Currently, CCTA is firmly established as a key non-invasive imaging modality for diagnosing CAD. It offers an excellent negative predictive value compared to other non-invasive imaging techniques. However, it is limitated artifacts and image noise, particularly in cases of severe coronary sclerosis or in patients with previously implanted stents [2].

The introduction of the novel photon-counting detector (PCD) marked a technological breakthrough [3]. Compared to conventional computed tomography (CT) systems, PCD-CT offers a threefold increase in spatial resolution while maintaining high temporal resolution. Previous studies have demonstrated that such ultrahigh spatial resolution PCD-CT reconstructions lead to improved image quality [4–6] and diagnostic accuracy compared to conventional CT, leading to clinically relevant improvements in risk classification in up to 50% of patients with CAD and a reduction in the discrepancy between CT-based and invasive coronary angiography assessments [7]. These effects were also validated in multicenter settings [8].

Despite these successes and advanced scanner-specific iterative reconstruction algorithms, increased image noise in ultrahigh spatial resolution can limit confidence in vessel-wall contour assessment and complicate stenosis measurement [1]. This is where DL-based denoising models, trained on dual-energy data, promise to simultaneously boost iodine contrast and reduce image noise [9–11]. Initial clinical applications show encouraging improvements, but the impact of these techniques on accurate anatomical detail remains insufficiently studied. This study, therefore, aimed to investigate the influence of a DL-based denoising model with simultaneous enhancement of iodine-based contrast attenuation for ultrahigh spatial resolution CCTA in patients with CAD.

## Materials and methods

### Study sample

This retrospective, single-centre study received approval from the local Ethics Committee (approval number 2022-16359) and is in accordance with the 2008 version of the Declaration of Helsinki. We retrospectively identified consecutive patients with stable chest pain who had been referred for CCTA to exclude CAD or the progression of known CAD and had received UHR-CCTA on a PCD-CT based on a coronary artery calcium score-based threshold (≥ 10).

### Sample size calculation and power analysis

An a priori power analysis was performed using GPower 3.1 [12, 13]. Effect sizes were derived from a previously published study that employed the same DL-based reconstruction tool and reported standardized differences in signal-to-noise ratio (SNR), contrast-to-noise ratio (CNR), and coronary wall sharpness (slope) between conventional and DL–enhanced reconstructions [11]. Based on these data, effect sizes (dz) of 2.08 for SNR, 2.11 for CNR, and 2.80 for slope were entered into GPower for a Wilcoxon signed-rank test for matched pairs (asymptotic relative efficiency method), assuming a normal parent distribution, two-tailed testing, an alpha level of 0.05, and a desired power (1–β) of 0.95. This a priori analysis yielded minimum required sample sizes of 6 subjects for SNR and CNR and 5 subjects for slope.

To account for potential exclusions due to motion artifacts or challenging examination conditions, as well as potential inflation of effect sizes derived from small previous studies, we prospectively enrolled a substantially larger cohort of 25 patients. *Post hoc* power calculations confirmed that the achieved power for all primary image quality parameters (SNR, CNR, and slope) exceeded 0.97 under the same assumptions, ensuring sufficient statistical power to detect clinically relevant differences between reconstruction methods.

### Imaging protocol

All CT scans were acquired using a first-generation PCD-CT system (NAEOTOM Alpha; Siemens Healthineers, Forchheim, Germany) containing two photon-counting cadmium-telluride detectors (collimation: 144 × 0.4 mm). Tube voltage was set to 120 kVp, and the gantry rotation time was 0.25 s. Scans were acquired in the ‘ultrahigh resolution’ mode (collimation 120 × 0.2 mm). Patients received 0.8 mg sublingual nitroglycerin approximately 5 min before the scan. If the patient’s heart rate exceeded 70 beats per minute, up to 25 mg (10.2 ± 7.0 mg, mean ± standard deviation (SD)) intravenous metoprolol was administered when clinically appropriate. This heart rate threshold also guided the choice of scan mode: sequential scanning with prospective end-diastolic electocardiographic triggering was used for heart rates below 70 bpm; an extended prospective triggering window was employed for heart rates above 70 beats per minute without arrhythmias; and spiral scanning with retrospective electocardiographic gating was used in patients with arrhythmias. To optimize contrast timing, a test bolus was administered prior to the diagnostic scan, using a biphasic injection protocol consisting of a continuous injection rate of 5 mL/s for 70 mL of iopromide (370 mg/mL, Ultravist 370, Bayer AG, Leverkusen, Germany), followed by a 25-mL saline flush.

All scans were reconstructed either directly on a dedicated off-site workstation at 0.2 slice thickness and 0.1 mm increments with vascular reconstruction kernels (Bv64). All datasets were reconstructed without iterative reconstruction, similar to a filtered-back projection reconstruction (Q_off_), and at each available level of vendor-specific iterative reconstruction (quantum iterative reconstruction, QIR) strength (Q_1–4_).

### DL-based denoising and iodine contrast enhancement

All CCTA Digital Imaging and Communications in Medicine–DICOM datasets were subsequently processed using a vendor-agnostic DL-based solution (ClariACE, ClariPi, Seoul, South Korea) for denoising and contrast enhancement. The model generates contrast-augmented images by scaling predicted iodine component pixel intensities using a user-defined boosting strength, then adding the scaled image to the input [11]. User-adjustable denoising strength was available. User-defined settings, including denoising strength, edge bending, and contrast enhancement (each ranging from 0 (no modification) to 1 (maximum effect)), were systematically tested in multiple combinations and adapted in consensus by two experienced readers (S.S., 5 years of experience in imaging; M.H., 7 years of experience in imaging). The final agreed settings that provided the best overall vessel assessability were denoising strength 1, edge bending 1, and contrast enhancement 1.

### Quantitative evaluation

Images were exported from the local picture archiving and communications system (Sectra Workstation; Linköping, Sweden) to a MATLAB-equipped workstation (R2022b, The MathWorks, Natick, MA, USA) for quantitative analysis. Data were blinded and interpreted by a board-certified radiologist with 1 year of experience in vascular imaging (A.G.). SNR, CNR, and coronary wall sharpness were assessed within a predefined rectangular region of interest (ROI) with a mean size of 991.25 ± 399.51 voxels (mean ± SD), corresponding to 171.53 ± 75.53 mm^3^ containing the vessel and adjacent perivascular tissue. The ROI was identically copied across Q_off_ and Q_1–4_ reconstructions (with and without DL) of the same patient to ensure direct comparability.

### Coronary wall sharpness

Coronary wall sharpness was assessed by means of the steepness (slope) of the grayscale transition curve between the right coronary artery (RCA) and the perivascular tissue. Measurements were performed on a perpendicular cross-section of the RCA in segment 2, with manually drawn luminal ROIs of consistent size across reconstructions; calcified plaques were carefully avoided during ROI placement. The vessel lumen and its center were automatically detected on manually drawn ROI on a transverse slice of the CCTA. Radial slope profiles originating at the vessel center were automatically traced through each vessel voxel to the edge. Image coordinates were transformed from Cartesian to a radial coordinate system. During this step, resampling to half the original voxel size (~ 0.2 mm; native voxel size ~ 0.4 × 0.4 × mm^3^) was performed using bicubic interpolation. For each voxel along the vessel circumference, one radial profile was generated. The total number of profiles therefore depended on vessel circumference and voxel size.

The steepest slope of each profile was determined using stepwise linear regression over 5 consecutive data points, corresponding to approximately 1 mm (~ 3 native voxels).

To reduce the influence of partial-volume effects (*e.g*., in non-perfectly perpendicular slices), only the 20% of profiles with the highest slope values were included in the final analysis. This empirically defined threshold was chosen to preferentially select profiles least affected by oblique sampling and partial-volume averaging. The median of these steepest slopes was used as the sharpness metric.

Upper and lower baseline signal intensities, required for SNR and CNR calculation, were derived from the same radial profiles. Baselines were determined using a sliding window of 5 data points along each profile. For the upper baseline, the window was shifted until the subsequent point fell below mean - SD of the window. The lower baseline was determined analogously in the reverse direction. This approach was adapted from previously published methodology [14, 15].

### Signal-to-noise ratio and contrast-to-noise ratio

SNR and CNR were assessed within the same rectangular ROI used for sharpness analysis to ensure spatial consistency.

The ROI was sampled using a quadratic sliding window of 5 × 5 mm^2^. Windows containing tissue transitions (as identified during slope analysis) were excluded from noise calculation.

Within each window, a second-order two-dimensional polynomial fit was applied and subtracted from the original gray values (detrending). This step suppresses low-frequency intensity variations and reduces the influence of tissue inhomogeneity. For small window sizes, this approach is comparable to mean subtraction but additionally accounts for gradual background trends. Alternative approaches (*e.g*., boxcar filtering) were tested during pipeline development and showed no relevant differences.

Noise was calculated as the SD of the detrended gray values within each window. The minimum SD across all valid window positions was used as the noise estimate. This approach was intentionally chosen because the ROI contains heterogeneous tissue rather than homogeneous reference material (*e.g*., air), and larger SD values may reflect structural heterogeneity rather than image noise.

It should, however, be acknowledged that selecting the minimum SD from multiple sliding windows may systematically underestimate the overall noise level compared to alternative approaches, such as the histogram-mode method, as previously shown by Anam et al [16]. Furthermore, differences in noise texture (*i.e*., the spatial frequency characteristics of the noise distribution) may also influence SNR and CNR values. Consequently, the absolute SNR and CNR values reported here may not be directly comparable to those derived from studies using different noise-estimation methodologies. As identical ROIs and noise-calculation procedures were applied across all reconstructions within each patient, relative comparisons between reconstruction methods remain internally consistent.

SNR was calculated as the upper baseline signal intensity divided by the noise value. CNR was calculated as the difference between the upper and lower baseline signal intensity divided by the noise value.

### Qualitative evaluation

To evaluate the image quality of Q_off_-Q_4_, three readers (A.Ki., M.H., and S.S., with 3, 7, and 5 years of imaging experience, respectively) were blinded to patient information and reconstruction type. Each independently assessed image sharpness, noise levels, vessel wall contours, and overall image quality using a 5-point Likert scale, with higher scores indicating higher image quality.

### Statistics

Statistical analysis was performed using R Statistics (Version 4.1.3, R Foundation, Vienna, Austria) and SPSS (Version 28, IBM Corp., Armonk, NY, USA). The Shapiro–Wilk test was used to assess the normality of data. Continuous variables are given as mean and standard deviation if normally distributed and as median/IQR in case of a non-normal distribution.

Ordinal variables were compared by analyzing the distribution of Likert-scale values. The Wilcoxon signed-rank test was used to compare variables. To evaluate the impact of QIR-strength level and additional deep-learning-based denoising on image quality metrics, linear mixed-effects models (LMM) were employed. The models included QIR level, reconstruction method, and their interaction as fixed effects, with individual patients treated as a random effect to account for intra-subject correlations. Degrees of freedom were estimated using the Satterthwaite approximation. *Post hoc* pairwise comparisons were performed using estimated marginal means (EMMs) with Tukey’s adjustment for multiple comparisons. Cronbach’s α was used to assess interrater consistency, with α-values of 0.5–0.6 considered poor agreement, 0.6–0.7 acceptable agreement, 0.7–0.9 good agreement, and values above 0.9 considered excellent agreement [17].

The *p*-values lower than 0.05 were considered statistically significant.

## Results

### Patient characteristics

A total of 25 patients were included in the study (age 65.6 ± 10.6 years, mean ± SD; 15 men, 10 women). Complete clinical datasets were available for all participants. A detailed summary of patient characteristics, including body mass index, median coronary artery calcium score, mean heart rate, metoprolol and sublingual nitrate application, as well as median CT dose index and dose-length product, is provided in Table 1.

**Table 1:** Patient characteristics

Age (years)	65.6 ± 10.6
Gender (female)	10/25 (40%)
Body mass index (kg/m^2^)	25.9 ± 3.6
Weight (kg)	77.4 ± 16.0
Coronary artery calcium score	446 [85–1,639]
Sublingual nitrate	25/25 (100%)
Intravenous metoprolol	23/25 (92%)
Computed tomography dose index (mGy)	9.5 [8.8–13.0]
Dose-length product (mGy × cm)	170 [160–196]
Heart rate (bpm)	63 ± 6

All data are reported as mean ± standard deviation or median with [interquartile ranges], depending on data distribution

### Qualitative evaluation

All readers rated overall image quality significantly higher for DL-CCTA than for conventional UHR-CCTA (4 [IQR, 4–5] *versus* 3 [IQR, 2–3]; *p* < 0.001). Deep-learning post-processed images reconstructed at the highest vendor-specific iterative reconstruction level (Q_4_) demonstrated the best results, with a median overall image quality score of 5 (IQR, 5–5).

**Fig. 1:**
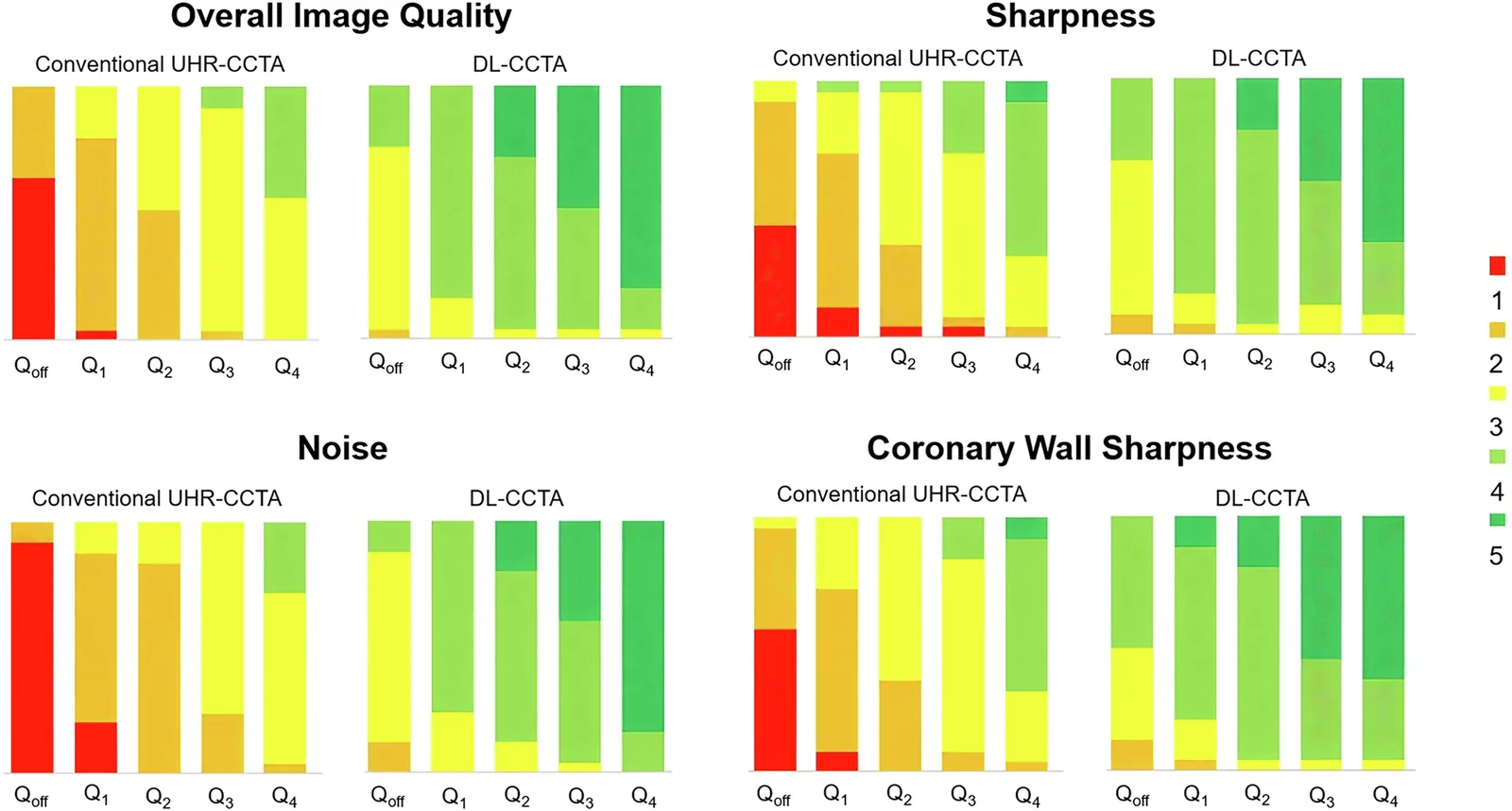
Bar charts representing the ratings for overall image quality, sharpness, noise, and vessel-wall contour with and without deep learning denoising for QIR levels 0–4

Image sharpness, noise level, and vessel wall contour delineation were all rated significantly better for DL-CCTA compared to conventional UHR-CCTA (sharpness, 4 [IQR, 4–5] *versus* 3 [IQR, 3–3]; noise, 4 [IQR, 4–5] *versus* 2 [IQR, 2–3]; vessel wall contour, 4 [IQR, 4–5] *versus* 3 [IQR, 2–3]; all *p* < 0.001). Representative median ratings for the qualitative parameters across all QIR levels are illustrated in Fig. 1.

The highest image quality was achieved for post-processed reconstructions at Q_4_, with median ratings of 5 (IQR, 4–5) for sharpness, 5 (IQR, 5–5) for noise, and 5 (IQR, 4–5) for vessel wall contour. There was no significant difference between vessel wall contour values of DL-CCTA values of Q_3_ and Q_4_ (*p* = 0.564), with both parameters displaying significantly higher values than DL-CCTA vessel wall contour values of Q_2_ and UHR-CCTA vessel wall contour values on all QIR levels (*p* = 0.013 and 0.005 or < 0.001, respectively). DL-CCTA overall image quality, sharpness, and noise Likert scores at Q_4_ were significantly higher compared to all other image sets (all *p* < 0.035).

Median ratings for all parameters across all QIR levels for both conventional UHR-CCTA and DL-CCTA are summarized in Table 2. The *p*-values of pairwise comparison of all parameters across all QIR levels are displayed in Table S1.

**Table 2:** Median subjective image quality ratings of the three readers on a 5-point Likert scale with [interquartile ranges]

	Image quality		Sharpness		Noise		Vessel-wall	
QIR	UHR-CCTA	DL-CCTA	UHR-CCTA	DL-CCTA	UHR-CCTA	DL-CCTA	UHR-CCTA	DL-CCTA
Q_off_	1 [1, 2]	3 [3, 4]	2 [1, 2]	3 [3, 4]	1 [1]	3 [3]	1 [1, 2]	4 [4]
Q_1_	2 [2]	4 [4]	2 [2, 3]	4 [4]	2 [2]	4 [4]	2 [2, 3]	4 [4]
Q_2_	2 [2, 3]	4 [4, 5]	2 [2, 3]	4 [4]	2 [2]	4 [4]	3 [2, 3]	4 [4]
Q_3_	3 [3]	4 [4, 5]	3 [3, 4]	4 [4, 5]	3 [3]	4 [4, 5]	3 [3]	4 [4, 5]
Q_4_	3 [3, 4]	5 [5]	4 [3, 4]	5 [4, 5]	4 [4]	5 [5]	4 [3, 4]	5 [4, 5]
Q_all_	3 [2, 3]	4 [4, 5]	3 [3]	4 [4, 5]	2 [2, 3]	4 [4, 5]	3 [2, 3]	4 [4, 5]

*CCTA* Coronary computed tomography angiography, *DL-CCTA* Deep learning-enhanced ultrahigh resolution photon-counting detector CCTA, *UHR-CCTA* Conventional ultrahigh resolution photon-counting detector CCTA, All *p* < 0.001

Interreader agreement ranged from acceptable to good, with comparable results across the conventional UHR-CCTA and DL-CCTA datasets.

### Quantitative evaluation

SNR and CNR were significantly higher in DL–denoised image sets compared with the original image sets at all QIR levels (*p* < 0.001 for both parameters across all levels). The highest SNR and CNR values were observed for DL–denoised images at Q_4_ compared to conventional CCTA (SNR: 258.0 ± 147.5 [mean ± SD] *versus* 145.2 ± 50.1, *p* < 0.001; CNR: 25.8 ± 17.1 *versus* 11.1 ± 4.5, *p* < 0.001).

A linear mixed-effects model revealed a significant effect of noise level on both SNR (F = 21.89, *p* < 0.001) and CNR (F = 13.88, *p* < 0.001) and a highly significant effect of deep-learning enhancement (F = 128.57, *p* < 0.001; F = 176.66, *p* < 0.001, respectively). No significant interaction between QIR level and DL denoising was detected for both SNR (F = 1.11, *p* = 0.354) and CNR (F = 1.63, *p* = 0.169), suggesting that the effect of DL on SNR and CNR was consistent across all reconstruction strength levels.

There was no significant difference in coronary wall sharpness values between DL-CCTA and conventional UHR-CCTA at Q_0_ (1,039 ± 244 *versus* 1,012 ± 477 mm/HU, *p* = 0.813). At all other QIR levels, coronary wall sharpness values were significantly higher for deep learning–denoised images than for the original reconstructions (all *p* < 0.027). The linear mixed effect model demonstrated that QIR level alone had no significant effect on coronary wall sharpness (F = 0.07, *p* = 0.99). In contrast, application of DL denoising significantly affected coronary wall sharpness (F = 40.29, *p* < 0.001). A significant interaction between QIR level and DL denoising was observed (F = 3.79, *p* = 0.006), indicating that the effect of DL varied depending on the QIR level.

Detailed SNR, CNR, and coronary wall sharpness values for both image types across QIR levels are summarized in Table 3. *p*-values of pairwise comparison of SNR, CNR, and coronary wall sharpness for both image types across all QIR levels are displayed in Table S2.

**Table 3:** SNR, CNR, and coronary wall sharpness for image sets with and without DL denoising

	SNR		CNR		Coronary wall sharpness (mm/HU)	
QIR	UHR-CCTA	DL-CCTA	UHR-CCTA	DL-CCTA	UHR-CCTA	DL-CCTA
Q_off_	48.4 ± 10.1	114.5 ± 38.2***	4.0 ± 1.6	12.8 ± 6.3***	1,039 ± 244	1,012 ± 477
Q_1_	64.7 ± 15.6	155.8 ± 56.7***	5.2 ± 1.8	16.1 ± 8.5***	906 ± 272	1,027 ± 550*
Q_2_	80.4 ± 20.3	181.9 ± 76.6***	6.4 ± 2.2	18.2 ± 9.6***	880 ± 351	1,063 ± 606**
Q_3_	101.7 ± 30.3	215.1 ± 96.7***	7.9 ± 3.1	22.0 ± 10.8***	883 ± 407	1,127 ± 605***
Q_4_	145.2 ± 50.1	258.0 ± 147.5***	11.1 ± 4.5	25.8 ± 17.1***	851 ± 281	1,118 ± 641**

Data are given as mean value ± standard deviation

*CCTA* Coronary computed tomography angiography, *CNR* Contrast-to-noise ratio, *DL-CCTA* Deep learning-enhanced ultrahigh resolution photon-counting detector CCTA, *QIR* Quantum iterative reconstruction, *SNR* Signal-to-noise ratio, *UHR-CCTA* Conventional ultrahigh resolution photon-counting detector CCTA

* *p* < 0.05, ** *p* < 0.01, *** *p* < 0.001

**Fig. 2:**
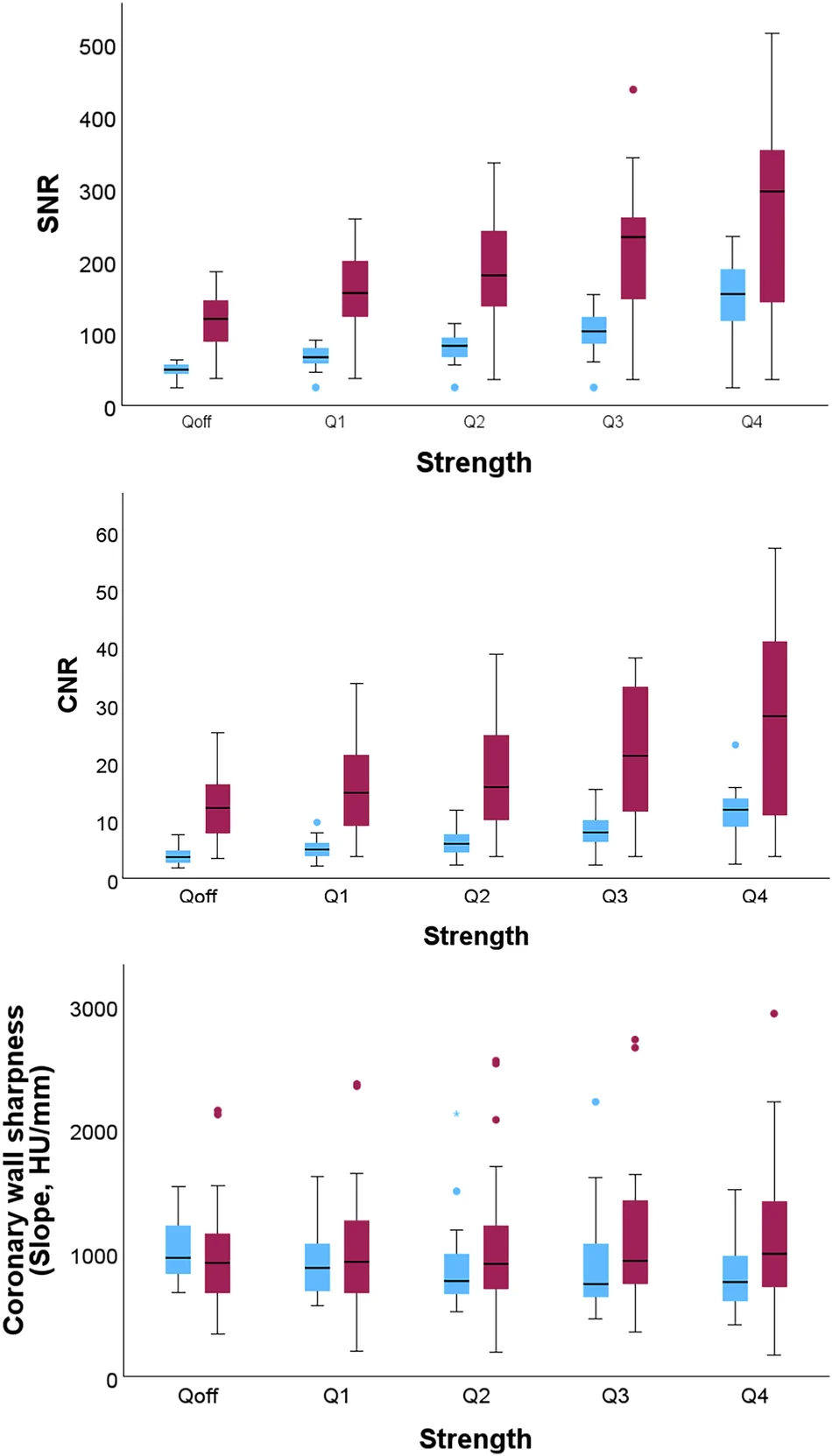
Box plots representing signal-to-noise ratio (SNR), contrast-to-noise ratio (CNR), and coronary wall sharpness without (blue) and with deep learning denoising (red) for every individual QIR strength level (Q_off_-Q_4_)

**Fig. 3:**
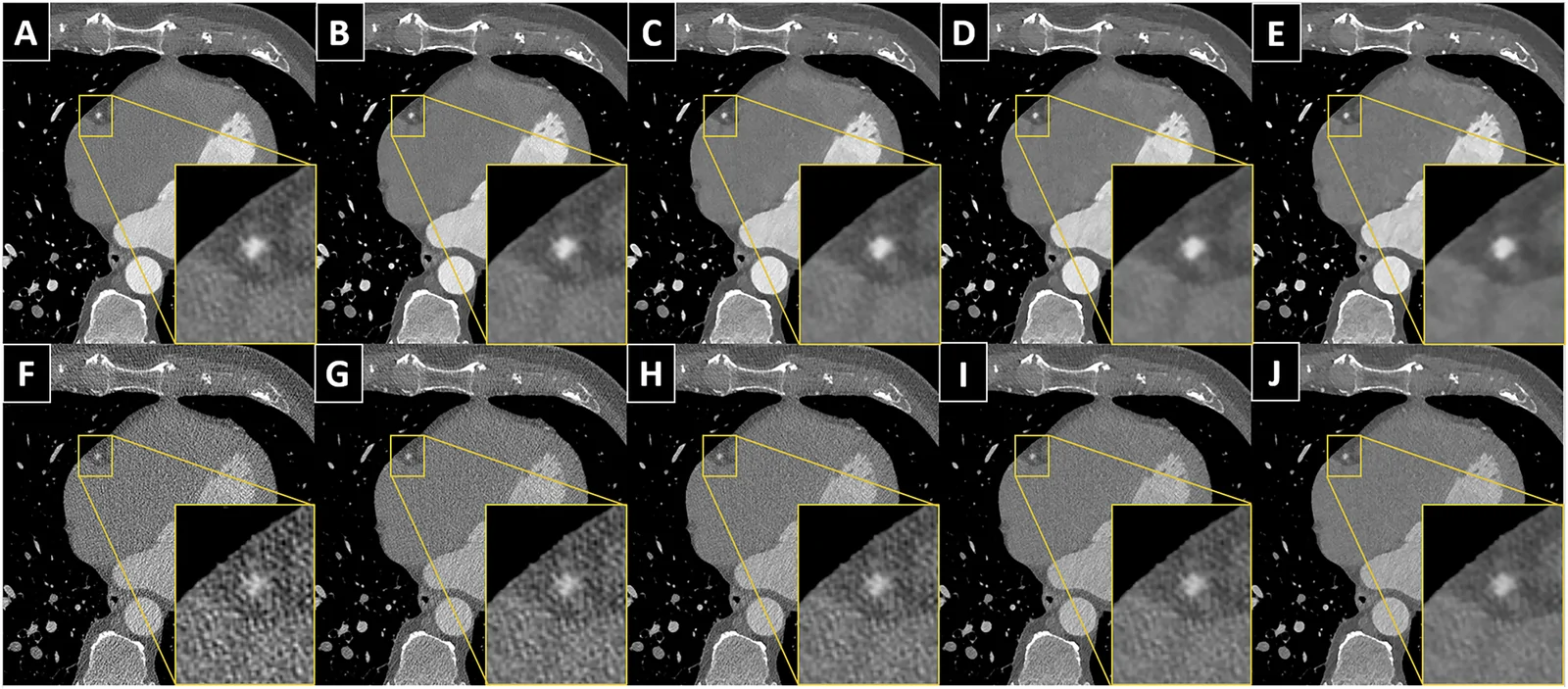
Axial cardiac images at the level of the right coronary artery with magnification of the regions of interest for sharpness measurements. The top row shows reconstructions with deep learning (DL) post-processing, while the bottom row shows those without. Columns from left to right show increasing levels of quantum iterative reconstructions (Q). **a**–**e** DL-UHR-PCD-CCTA; **f**–**j** UHR-PCD-CCTA; **a**, **f** Q_off_; **b**, **g** Q_1_; **c**, **h** Q_2_; **d**, **i** Q_3_; **e**, **j** Q_4_. CCTA, Coronary computed tomography; PCD, Photon-counting detector; UHR, Ultrahigh resolution

**Fig. 4:**
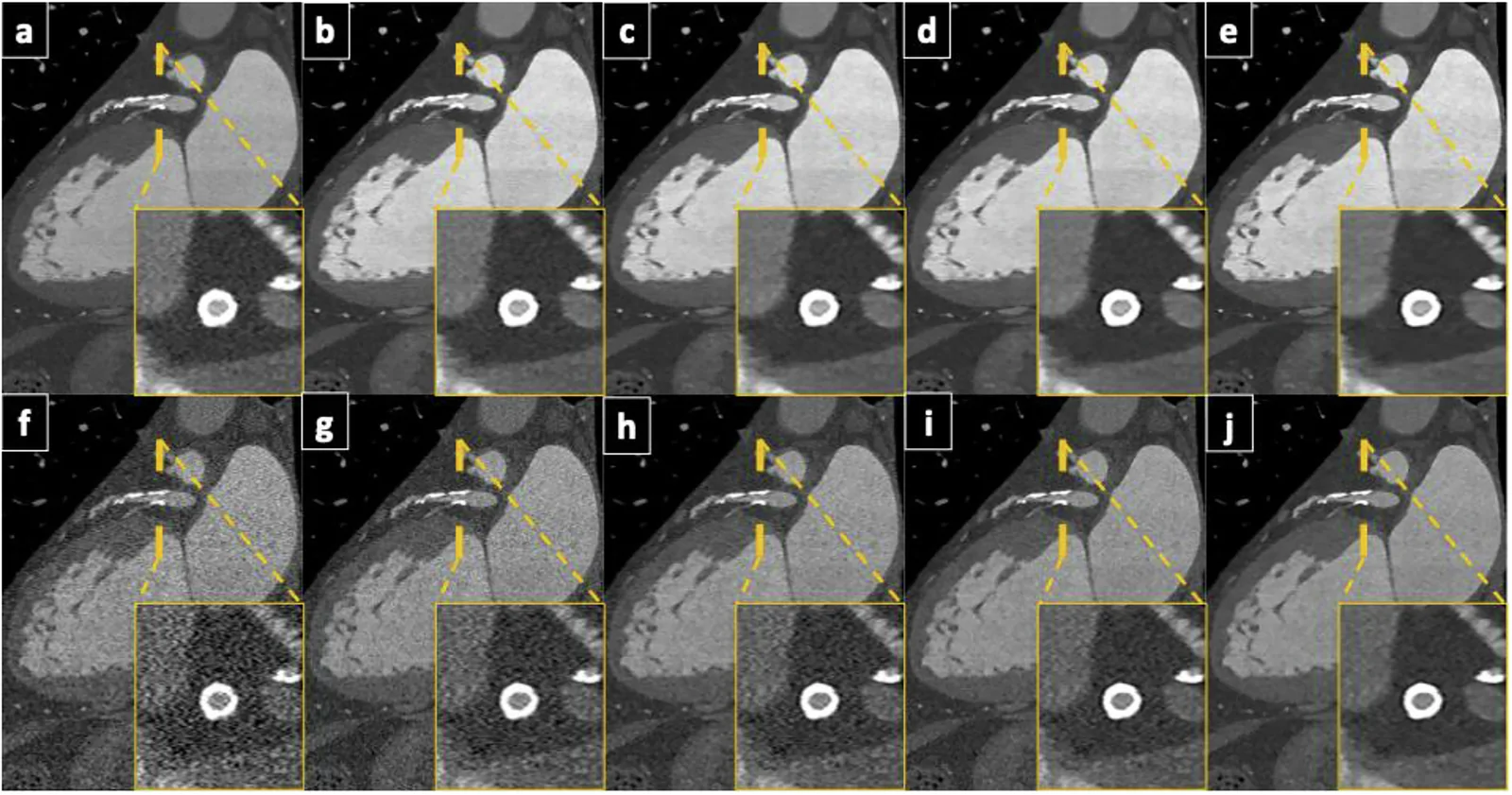
Sagittal cardiac images at the level of the right coronary artery with magnification of the regions of interest for sharpness measurements. The top row shows reconstructions with DL post-processing, while the bottom row shows those without. Columns from left to right show increasing levels of quantum iterative reconstructions. **a**–**e** DL-UHR-PCD-CCTA; **f**–**j** UHR-PCD-CCTA; **a**, **f** Q_off_; **b**, **g** Q_1_; **c**, **h** Q_2_; **d**, **i** Q_3_; **e**, **j** Q_4_. CCTA, Coronary computed tomography; PCD, Photon-counting detector; UHR, Ultrahigh resolution

SNR, CNR, and coronary wall sharpness distributions for both image sets across all QIR levels are illustrated by the box plots in Figs. 2–4 show axial and sagittal RCA images comparing DL *versus* non-DL reconstructions across the quantum iterative levels.

## Discussion

This study investigated whether DL denoising with simultaneous iodine contrast enhancement improves UHR-CCTA from PCD-CT, despite image noise remaining a limitation of ultrahigh spatial resolution scans. Our analyses showed that post-processed images achieved superior qualitative evaluation compared to conventional UHR-CCTA in all defined items. These results were also reflected in the quantitative evaluation, with significantly higher SNR and CNR values across all levels of vendor-specific iterative reconstruction strength, as well as improved coronary wall sharpness in all reconstructions except for filtered-back projections.

To our knowledge, this is the first study to demonstrate the benefits of DL denoising and iodine-based contrast enhancement in combination with UHR-CCTA on a PCD-CT system. Our results are consistent with previous studies on energy integrating detector CT systems and various non-cardiac anatomical regions [18–22]. For example, in abdominal imaging, DL reconstructions significantly improved the detectability of liver metastases [23, 24]. Deep learning reconstruction also demonstrates significantly better image quality and noise reduction in vascular imaging, for example, in aortic CT angiography [25] or neurovascular imaging for stroke diagnostics [11].

Additionally, numerous studies have demonstrated the robustness of DL denoising across a range of noise intensities in the cardiovascular field [26–28]. However, previous studies have typically investigated DL reconstructions at only one or two dose levels, not in cardiac ultrahigh spatial-resolution CT. Our results further extend these findings by demonstrating that the DL algorithm operates robustly and consistently across Q_off_–Q_4_, achieving significant noise reduction and delivering improved image quality.

As previously mentioned, the Q_off_ coronary wall sharpness values of the DL-CCTA were lower (albeit not statistically significant) than in the original images, although the DL images were qualitatively rated superior in all parameters. The most likely explanation for this is that DL reconstructions generally reduce image noise and produce a more homogeneous visual appearance. Consequently, the perivascular fat density values of individual voxels also converge, potentially affecting the analysis of coronary wall sharpness. A plausible explanation for this finding is that the readers are more accustomed to the image appearance of UHR-CCTA, whereas they are not yet familiar with that of DL-CCTA.

As the first study to apply DL denoising with simultaneous iodine contrast enhancement to UHR-CCTA from PCD-CT, our findings indicate substantial potential for further protocol optimization, including radiation dose reduction and lower contrast agent volumes in clinical coronary CT angiography.

This study has several limitations. First, the retrospective design may have introduced selection bias. Second, the limited sample size reduces the generalizability of our findings and precludes adequately powered subgroup analyses. In particular, the number of patients with relevant CAD, coronary calcifications, or distinct lesion morphologies was too small to allow reliable stratified analyses according to disease burden or plaque characteristics. Third, the assessment of coronary stenoses and plaque quantification was not a predefined endpoint of this study. Importantly, no systematic comparison with invasive coronary angiography as the reference standard was performed. Therefore, no conclusions can be drawn regarding the impact of the DL models on diagnostic accuracy for stenosis grading or on agreement with invasive findings. Similarly, we did not systematically compare DL-processed datasets with the original clinical CT reports. Consequently, the potential influence of DL post-processing on clinical decision-making, downstream management, or diagnostic reclassification remains unclear. The clinical relevance of the observed improvements in image quality metrics and subjective image assessment therefore requires further validation in outcome-oriented studies.

A further methodological limitation concerns our noise estimation: the minimum SD across multiple sliding windows was used to reduce the influence of tissue heterogeneity within heterogeneous perivascular ROIs. While this approach provided consistent and internally comparable measurements across all reconstructions within each patient, it may systematically underestimate the overall noise level and thereby produce higher absolute SNR and CNR values than other noise-estimation approaches (*e.g*., histogram-mode methods [16]).

In addition, differences in noise texture between reconstruction methods may also influence these metrics. Absolute SNR and CNR values should therefore not be directly compared to those reported in studies using different noise-calculation methodologies, although relative comparisons between reconstruction methods within this study remain valid.

In the subset of patients with coronary calcifications, the model demonstrated improved image quality. However, this must be interpreted with caution, as our study is clearly underpowered for robust subgroup evaluation. The number of patients with relevant coronary calcifications and significant CAD was too small to permit reliable subgroup analysis and conclusions regarding diagnostic performance. Importantly, the present investigation was designed as an exploratory study, primarily aiming to evaluate at which levels of vendor-specific iterative reconstruction DL-based post-processing provides the greatest technical benefit. It was not powered to assess diagnostic accuracy or clinical endpoints.

To address these questions more comprehensively, we are planning a larger, prospective study incorporating invasive coronary angiography as the reference standard. This follow-up investigation will specifically evaluate the diagnostic impact of DL-based denoising and contrast enhancement on stenosis grading, plaque characterization, and agreement with invasive findings, thereby clarifying the true clinical value of DL in patients with relevant CAD.

Another relevant aspect is the potential combination of different DL algorithms operating at various stages of image acquisition and post-processing (*i.e*., multi-algorithmic DL approaches). Emerging evidence suggests that hybrid strategies combining multiple algorithmic levels may outperform single-step approaches by leveraging complementary strengths and compensating for individual weaknesses. Future investigations should systematically analyze interactions between algorithms at different processing levels and evaluate their combined effect on image quality, diagnostic accuracy, and clinical relevance.

In conclusion, post-processing DL-based denoising and iodine-specific contrast enhancement models can mitigate the noise penalty associated with ultrahigh-resolution CCTA and simultaneously enhance anatomic detail. The most pronounced benefits were observed at the highest vendor-specific iterative reconstruction strength levels, suggesting incremental value of DL-based post-processing. However, the impact on diagnostic accuracy, clinical reporting, and patient management remains to be established in larger, prospective, outcome-driven studies.

## Supplementary information

**Additional file 1: Table S1:** Pair-wise comparison of median subjective image quality ratings. **Table S2:** Pair-wise comparison of SNR, CNR and coronary wall sharpness.

## Data Availability

The datasets generated and/or analyzed during the current study are not publicly available due to institutional and data protection regulations but are available from the corresponding author on reasonable request.

## Abbreviations

CAD: Coronary artery disease
CCTA: Coronary CT angiography
CNR: Contrast-to-noise ratio
CT: Computed tomography
DL: Deep learning
DL-CCTA: DL-based CCTA
IQR: Interquartile range
PCD-CT: Photon-counting detector CT
QIR: Quantum iterative reconstruction
RCA: Right coronary artery
ROI: Region of interest
SD: Standard deviation
SNR: Signal-to-noise ratio
UHR: Ultrahigh resolution
