# Supplementary material for: Deep learning denoising and deep learning–augmented contrast enhancement improve ultra-high-resolution photon-counting-detector CCTA image quality
Source: Eur Radiol Exp. 2026 Nov 13;10:181. doi: 10.1186/s41747-026-00809-9 (PMC13768433; doi:10.1186/s41747-026-00809-9)
Supplement: Supplementary file 1 [file 41747_2026_809_MOESM1_ESM.pdf]

Deep Learning Denoising and Deep Learning-Augmented Contrast Enhancement improve Ultra-High-Resolution Photon-Counting-Detector CCTA Image Quality

ELECTRONIC SUPPLEMENTARY MATERIAL

Eur Radiol Exp (2026) Steinmetz S, Kilburg A, Sanner A et al.

Table S1: Pair-wise comparison of median subjective image quality ratings

Image Quality

	Qoff	Q1	Q2	Q3	Q4	DL-Qoff	DL-Q1	DL-Q2	DL-Q3
Q1	<0.001								
Q2	<0.001	0.011							
Q3	<0.001	<0.001	<0.001						
Q4	<0.001	<0.001	<0.001	0.002					
DL-Qoff	<0.001	<0.001	<0.001	0.102	0.999				
DL-Q1	<0.001	<0.001	<0.001	<0.001	0.008	<0.001			
DL-Q2	<0.001	<0.001	<0.001	<0.001	<0.001	<0.001	0.004		
DL-Q3	<0.001	<0.001	<0.001	<0.001	<0.001	<0.001	<0.001	0.166	
DL-Q4	<0.001	<0.001	<0.001	<0.001	<0.001	<0.001	<0.001	0.002	0.011

Sharpness

	Qoff	Q1	Q2	Q3	Q4	DL-Qoff	DL-Q1	DL-Q2	DL-Q3
Q1	0.002								
Q2	<0.001	0.008							
Q3	<0.001	<0.001	0.001						
Q4	<0.001	<0.001	0.001	0.002					
DL-Qoff	<0.001	<0.001	0.002	0.803	0.999				
DL-Q1	<0.001	<0.001	<0.001	<0.001	0.527	<0.001			
DL-Q2	<0.001	<0.001	<0.001	<0.001	<0.001	<0.001	0.014		
DL-Q3	<0.001	<0.001	<0.001	<0.001	0.003	<0.001	0.005	0.366	
DL-Q4	<0.001	<0.001	<0.001	<0.001	<0.001	<0.001	<0.001	0.008	0.035

Noise

	Qoff	Q1	Q2	Q3	Q4	DL-Qoff	DL-Q1	DL-Q2	DL-Q3
Q1	<0.001								
Q2	<0.001	0.014							

Q3	<0.001	<0.001	<0.001						
Q4	<0.001	<0.001	<0.001	0.003					
DL-Qoff	<0.001	<0.001	<0.001	0.034	0.917				
DL-Q1	<0.001	<0.001	<0.001	<0.001	<0.001	<0.001			
DL-Q2	<0.001	<0.001	<0.001	<0.001	<0.001	<0.001	0.021		
DL-Q3	<0.001	<0.001	<0.001	<0.001	<0.001	<0.001	<0.001	0.052	
DL-Q4	<0.001	<0.001	<0.001	<0.001	<0.001	<0.001	<0.001	<0.001	0.003
**Vessel-Wall**									
	Qoff	Q1	Q2	Q3	Q4	DL-Qoff	DL-Q1	DL-Q2	DL-Q3
Q1	<0.001								
Q2	<0.001	<0.001							
Q3	<0.001	<0.001	<0.001						
Q4	<0.001	<0.001	<0.001	<0.001					
DL-Qoff	<0.001	<0.001	<0.001	0.033	0.967				
DL-Q1	<0.001	<0.001	<0.001	<0.001	0.317	0.005			
DL-Q2	<0.001	<0.001	<0.001	<0.001	0.008	<0.001	0.052		
DL-Q3	<0.001	<0.001	<0.001	<0.001	<0.001	<0.001	<0.001	0.013	
DL-Q4	<0.001	<0.001	<0.001	<0.001	<0.001	<0.001	<0.001	0.005	0.564

p-Values of pair-wise Wilcoxon signed Rank test row> column

Table S2: Pair-wise comparison of SNR, CNR and coronary wall sharpness

Coronary wall sharpness

	Qoff	Q1	Q2	Q3	Q4	DL-Qoff	DL-Q1	DL-Q2	DL-Q3
Q1	0.999								
Q2	0.999	0.999							
Q3	0.999	0.999	0.999						
Q4	0.999	0.999	0.999	0.999					
DL-Qoff	0.999	0.045	0.009	0.011	0.013				
DL-Q1	0.573	0.006	0.002	0.001	0.002	0.002			
DL-Q2	0.376	0.008	0.005	0.002	0.009	0.013	0.057		
DL-Q3	0.293	0.008	0.003	0.002	0.008	0.008	0.010	0.097	
DL-Q4	0.179	0.005	0.002	0.002	0.005	0.006	0.018	0.054	0.018

CNR

	Qoff	Q1	Q2	Q3	Q4	DL-Qoff	DL-Q1	DL-Q2	DL-Q3
Q1	<0.001								
Q2	<0.001	<0.001							
Q3	<0.001	<0.001	0.001						
Q4	<0.001	<0.001	<0.001	<0.001					
DL-Qoff	<0.001	<0.001	<0.001	<0.001	0.011				
DL-Q1	<0.001	<0.001	<0.001	<0.001	<0.001	<0.001			
DL-Q2	<0.001	<0.001	<0.001	<0.001	0.002	0.002	0.010		
DL-Q3	<0.001	<0.001	<0.001	<0.001	<0.001	<0.001	<0.001	<0.001	
DL-Q4	<0.001	<0.001	<0.001	<0.001	<0.001	<0.001	0.006	<0.001	0.013

SNR

	Qoff	Q1	Q2	Q3	Q4	DL-Qoff	DL-Q1	DL-Q2	DL-Q3
Q1	<0.001								
Q2	<0.001	<0.001							

Q3	<0.001	<0.001	<0.001						
Q4	<0.001	<0.001	<0.001	<0.001					
DL-Qoff	<0.001	<0.001	<0.001	0.013	0.999				
DL-Q1	<0.001	<0.001	<0.001	<0.001	0.046	<0.001			
DL-Q2	<0.001	<0.001	<0.001	<0.001	0.031	<0.001	0.005		
DL-Q3	<0.001	<0.001	<0.001	<0.001	0.001	<0.001	<0.001	<0.001	
DL-Q4	<0.001	<0.001	<0.001	<0.001	0.002	<0.001	0.002	<0.001	0.008

p-Values of pair-wise Wilcoxon signed Rank test row> column
